# Testing the own-age bias in face recognition among younger and older adults via the face inversion effect

**DOI:** 10.1177/03010066251405714

**Published:** 2025-12-15

**Authors:** Ciro Civile, Guangtong Wang

**Affiliations:** 1 Department of Psychology, Faculty of Health & Life Sciences, University of Exeter, Exeter, UK

**Keywords:** own-age bias (OAB), face inversion effect, face recognition, old/new recognition task

## Abstract

This study examines the perceptual expertise basis of the own-age bias (OAB)—better recognition of faces from one's own age group—in young (n = 64; 19–30 years) and older adults (n = 64; 69–80 years). Participants engaged in an old/new recognition task involving upright and inverted young and older faces. The results revealed a robust OAB in the younger group, characterized by a reduced face inversion effect (“FIE”)—more accurate recognition for upright versus inverted faces—when processing older/other-age faces compared to the pronounced FIE for own/younger-age faces. This difference was primarily driven by disrupted performance for upright older faces vs. upright young faces. In contrast, no OAB was observed in the older group, which exhibited a strong FIE for both own/older- and other/younger-age faces, with upright older faces being recognized more accurately than in the younger group. These findings underscore the importance of perceptual expertise in explaining the OAB.

## Introduction

Numerous studies in the face recognition literature have demonstrated that young adults exhibit enhanced recognition of faces within their own age group compared to faces from other age groups—such as older adults or children—a phenomenon known as the own-age bias (“OAB”) (Anastasi & Rhodes, 2006; Cronin et al., 2021; Hills & Lewis, 2011; Wallis et al., 2012). Moreover, some studies have investigated the OAB in older adults, yielding contrasting results that suggest factors such as stimuli, experimental procedures, and the age range of participants influence the effects (Denkinger & Kinn, 2018; Proietti et al., 2019; Rhodes & Anastasi, 2012; Strickland-Hughes et al., 2020; Wiese et al., 2013).

A central debate regarding the OAB concerns its underlying nature—whether it arises from social motivations or perceptual expertise—paralleling the broader literature on “own” biases in face recognition. The most extensively studied phenomenon in this context is the own-race bias (“ORB”) (Malpass & Kravitz, 1969; Meissner & Brigham, 2001).

Over time, various motivational accounts linked to social categorization have been proposed to explain the ORB (Rodin, 1987; Wegner & Bargh, 1998). The most comprehensive recent explanation suggests that the ORB reflects a tendency for individuals to think categorically about outgroup members, leading to different processing of facial features compared to ingroup members (Hugenberg et al., 2013; Levin, 2000). Ingroup faces (e.g., own-race) prompt perceivers to focus on distinguishing features within their group, whereas outgroup faces (e.g., other race) are often categorized based on prototypical features—such as race, sex, or age—that are common across all outgroup faces, complicating individual differentiation (Young et al., 2012). Therefore, it is argued that social categorizations influence how facial features are used for recognition, and this process similarly applies to categories like sex and age, thus illuminating the OAB.

An example of this is recent work conducted by Rollins et al. (2020), in which young adult participants (age range 18–23) engaged in an old/new recognition task commonly used in face recognition research. During the experiment, participants first completed a study/encoding phase, in which they were asked to memorize a set of faces belonging to their own age group (young adults) or another age group (children). Subsequently, they completed a recognition task that showcased the same face images from the study phase, intermixed with new faces. The critical manipulation involved the instructions given during the study phase. The authors found that when participants were instructed to provide verbal judgments about the age of the faces, the recognition results revealed a robust OAB, with better recognition for own-age faces compared to others. However, this bias was reduced—though still present—when participants were instead instructed to make a gender judgment about the faces during the study phase. Although these findings are interesting, they do not definitively establish a motivational account. The reduced OAB observed could simply be due to the sex-judgment instructions acting as a distractor, making face memorization more difficult for participants, as also indicated by the decreased performance for own-age faces in the sex-judgment condition.

Alternatively, cognitive scientists have proposed a perceptual expertise account, suggesting that the ORB results from limited visual experience with faces of other races, leading to diminished perceptual expertise. Evidence has shown that the magnitude of the ORB correlates with the level of interracial contact individuals experience in daily life (Zhao et al., 2014). Concurrently, research on the OAB involving children revealed that 3-year-old children are more accurate at recognizing young adult faces than both newborns and their own age group. Although not directly tested, the authors suggested that this lack of an OAB likely reflects increased contact with adult faces during early childhood and the developmental task of forming attachments to their primary caregivers, typically young adults. This, in turn, supports a perceptual expertise-based explanation for the effect (Cassia et al., 2012; Cassia et al., 2009). Several related follow-up studies have further provided evidence that the OAB diminishes among those who have more frequent contact with other age groups, such as individuals who work with children or infants (Harrison & Hole, 2009), and those with varying levels of exposure to different age groups in their daily lives (Wiese, 2012; Wiese et al., 2013). Although the precise mechanism by which contact improves facial recognition remains unclear, a prominent explanation derived from the ORB literature suggests that experience with a specific group enhances perceptual expertise. This allows for more detailed mental representations of facial features (e.g., Valentine, 1991). Since facial features can vary across multiple dimensions—such as shape, size, and spatial relationships—more experience may enable individuals to better detect and encode subtle differences that a naïve observer might not recognize as salient.

A widely used paradigm to test the perceptual expertise hypothesis in face recognition research is the face inversion effect (FIE) (Diamond & Carey, 1986; Valentine, 1988; Yin, 1969). The FIE refers to a decline in recognition performance when faces are presented upside down. Research suggests that inversion disrupts our expertise in processing configural/holistic information—the spatial relationships among the main features of a face—resulting in reduced recognition (Gauthier & Tarr, 1997; Maurer et al., 2002; McLaren, 1997). This paradigm is one of the most frequently employed in the field and is regarded as a key indicator of perceptual expertise, supported by the demonstration that a robust inversion effect can be obtained with novel artificial stimuli—such as Greebles or checkerboards—that participants are familiarized with during the experiment (e.g., Civile, Zhao, et al., 2014; Civile et al., 2024; Gauthier & Tarr, 1997; McLaren & Civile, 2011). Importantly, Civile, Zhao, et al., (2014), demonstrated that a strong inversion effect is observed for checkerboard stimuli drawn from a prototype-defined category, which participants were familiarized with, sharing a common configuration. Conversely, no inversion effect was observed for checkerboard stimuli that did not share such a configuration. Several studies in the literature have also shown that manipulating facial configural or holistic information—through techniques such as Thatcherization, scrambling, or Mooney manipulations—leads to a reduction in the FIE, manifested as decreased performance for upright faces (Civile, McLaren, et al., 2014; Civile et al., 2016; Latinus & Taylor, 2005; McCourt et al., 2023; Schwiedrzik et al., 2018). Taken together, these findings suggest that inversion impairs our ability to utilize the spatial relationships among the main features within a face—the configural and holistic information—thereby disrupting recognition performance. Because we are not trained to use configural and holistic cues when faces are presented upside down, our recognition performance is reduced.

Importantly, the FIE has been used to investigate the nature of the ORB. For instance, Vizioli et al. (2010) observed a reduced FIE for other-race faces compared to own-race faces, primarily due to impaired recognition of upright other-race faces. More recently, Civile and McLaren (2022) employed transcranial Direct Current Stimulation (tDCS) to explore whether the ORB could be diminished by disrupting perceptual expertise. In their study, Western Caucasian participants were assigned to either an anodal or a sham/control condition before completing an old/new recognition task—commonly used in FIE research—involving both upright and inverted Western Caucasian and East Asian faces. The sham group confirmed a robust ORB, characterized by a smaller FIE for other-race faces—due to poorer recognition of upright other-race faces. Importantly, under the anodal tDCS condition, the interaction indicating the ORB was eliminated, as the FIE for own-race faces was reduced to the level seen for other-race faces, stemming from decreased recognition performance for upright own-race faces. This demonstrates that disrupting perceptual expertise can effectively mitigate the ORB, marking a significant advancement in understanding the mechanisms behind this bias.

As with the ORB, a few studies have used the FIE to investigate perceptual expertise mechanisms at the basis of the OAB. For instance, Kuefner et al. (2008) demonstrated that young adults exhibited enhanced recognition of upright own age faces relative to both newborn and child faces; however, this own-age advantage disappeared when the faces were presented in an inverted orientation. In another experiment, Cassia et al. (2009) found a similar FIE for newborn and adult faces among nurses working in a maternity ward—individuals with substantial expertise in seeing newborn faces—while control participants (novices) did not exhibit an FIE for newborn faces. Collectively, these studies suggest that the FIE is reduced for other-age faces when sufficient perceptual expertise has not been developed, with upright faces serving as a primary factor influencing the modulation of the FIE —findings consistent with those in the ORB literature. In the case of the ORB, the reduced recognition performance for upright other-race faces can be explained by differences in facial features across own- and other-race faces—that is, a lack of expertise in processing the configural/holistic information of those different features. In the OAB the fact that the FIE is diminished for other-age faces suggests that facial features may change with age, thereby influencing perceptual expertise in processing configural/holistic information.

More importantly, whereas the ORB includes individuals whose ingroup membership remains unchanged (they are members of a particular race throughout their lives), the OAB represents an exception to this. Aging necessitates that individuals who were once members of one group (e.g., young adults) become members of another (e.g., older adults). This progression suggests that individuals with opportunities to develop expertise with faces of other ages—by virtue of prior membership in those groups (e.g., older adults’ previous experience as young adults)—would be less likely to exhibit the OAB. Thus, it is reasonable to hypothesize that, for example, older individuals might not show a difference in the FIE when presented with own- and other-age (e.g., young adult) faces. However, no published research to date has directly investigated the OAB, as indexed by the FIE, in young and older adults to test the perceptual expertise hypothesis.

In this paper, we extend the OAB literature by examining this phenomenon in young adults aged 19–30 years and older adults aged 69–80 years. Based on predictions from the ORB literature (Civile & McLaren, 2022), we anticipate observing a robust OAB in the younger group, characterized by a reduced FIE for older/other-age faces compared to the pronounced FIE for own-age faces. This difference is expected to primarily result from disrupted recognition performance for upright older faces relative to upright young faces. Importantly, in the older group, we expect no OAB; a strong FIE should be evident for both own- and other-age faces, with upright older faces being recognized better than in the younger sample of participants. These findings would highlight the significance of perceptual expertise in face recognition.

## Method

### Participants

Overall, 128 naïve Western Caucasian subjects participated in the study. The participants were recruited through the online platform Prolific and received monetary compensation in accordance with the platform's fair policy. Sixty-four participants were assigned to the “younger” group, consisting of 32 females and 32 males; their mean age was 24.9 years, with an age range of 19–30 years. Other 64 participants were assigned to the “older” group, comprising 32 females and 32 males; their mean age was 72.7 years, with an age range of 69–80 years. All participants had normal or corrected-to-normal vision and were fluent in English.

All procedures were conducted in accordance with relevant guidelines and regulations, and were approved by the Psychology Research Ethics Committee at the University of Exeter.

The sample size was determined through a prior power analysis conducted using G*Power software (Faul et al., 2007) for the overall 2 × 2 × 2 design (Orientation [upright, inverted]×Face Age [younger, older]×Participant Age [younger, older]). The analysis indicated that this sample would provide over 0.80 power to detect a medium effect size (d = 0.5). Specifically, the information input into G*Power included a medium effect size indicated by f = 0.25, with two between-subjects groups (younger and older participants) and two measurements (Orientation×Face Age).

### Materials

The study utilized a set of high-resolution Western Caucasian faces from the FACES database (https://faces.mpdl.mpg.de/imeji). The set consists of images of naturalistic faces of young, middle-aged (although these were not used in our study), and older women and men. The original stimuli were created by recruiting face models with an “average” appearance, without prominent features such as beards, tattoos, or piercings. The models included 60 young individuals (M = 24.3 years, SD = 3.5; age range 19–30), 60 middle-aged individuals (M = 49.0 years, SD = 3.9; age range 39–55), and 58 older individuals (M = 73.2 years, SD = 2.8; age range 69+), all of Western Caucasian background.

These models were trained to mimic six main facial expressions (neutrality, sadness, disgust, fear, anger, and happiness), including phases for emotion induction (triggering spontaneous emotion) and controlled expression. High-quality digital photographs were then taken under consistent lighting conditions: 120° frontal lighting from above via a striplight for soft illumination, 220° brightening from below, with white balance set to neutral gray. All images were color photographs of models who directed their gaze toward the camera.

Following stimulus development, only the most prototypical pictures of the models (as rated by external coders) were selected for the validation study, in which 154 participants—comprising 52 young, 51 middle-aged, and 51 older individuals—rated the faces in terms of facial expression and perceived age. Based on this validation, authors created a specific subset of younger, middle-aged, and older faces (Ebner et al., 2010).

In follow-up studies, authors have used the same set of stimuli to investigate changes in attractiveness ratings—such as those influenced by participants’ emotional states (e.g., Ebner et al., 2018)—or face emotion recognition across different age groups (Holland et al., 2019) —or perceived trustworthiness across lifespan (Pehlivanoglu et al., 2023).

For our study, we used only the subset of neutral-expression images of younger (aged 19–30 years) and older individuals (aged 69–80 years), resulting in 56 images per group, with an equal number of male and female images. Inverted faces were generated by rotating the original images by 180 degrees. In total, 224 faces were used in the study: 112 upright faces and 112 inverted faces. Images were presented centrally on the screen (following a fixation cue) at a size of 5.5 × 7 cm.

### Behavioral Task

The old/new recognition task consisted of two parts: a ‘study phase’ and an ‘old/new recognition phase’ (Civile et al., 2014; Civile et al., 2016; Civile & McLaren, 2022).

In the **study phase**, each participant was shown 56 upright faces (28 younger and 28 older) and 56 inverted faces (28 younger and 28 older). The faces were presented one at a time in a randomized order, with no response required from the participant. In line with previous studies using the same task (Civile et al., 2014; Civile et al., 2016; Civile & McLaren, 2022), participants were instructed that a series of faces would appear one at a time and were encouraged to try to memorize them. Following the instructions, each trial began with a fixation cross displayed at the center of the screen for 1 s. This was followed by the presentation of a face stimulus for 3 s before moving to the next trial. After all, 112 faces had been shown, the program displayed instructions for the recognition task.

In the subsequent **recognition phase**, 112 novel faces (half upright and half inverted) were added to the 112 faces seen during the study phase. All 224 faces were presented sequentially in random order, and participants were asked to press the ‘.’ key if they recognized the face as having been shown during the study phase, or the ‘x’ key if they did not. The key assignments were counterbalanced across participant groups. Each trial began with a fixation cross displayed at the center of the screen for 1 s, followed by the presentation of a face stimulus for a maximum of 4 s, within which participants had to respond. If no response was made within this period, the trial timed out, a feedback trial saying “Too Slow” appeared and the next one began automatically.

For each participant, each face stimulus appeared in only one orientation during the experiment. Across different subject groups, each stimulus was presented in every condition—upright seen, inverted seen, upright not seen, and inverted not seen.

## Results

### Data Analysis

Our primary measure was performance accuracy in the old/new recognition task. Accuracy data were inspected for participants whose scores fell beyond ±3 SD standard deviations from the mean. No participants were excluded from the analysis. The data from all the participants in each experimental condition was used to compute a *d*-prime (*d’*) sensitivity measure (Stanislaw & Todorov, 1999) for the recognition task (old and new stimuli for each stimulus type) where a *d* = of 0 indicates chance-level performance.

In our Supplemental Material, we have also reported the analysis using percentage correct accuracy (%) to provide a clearer picture of behavioral performance across all conditions in both experiments. The analysis confirmed all the effects found with *d’*.

To calculate *d*’, we used subjects’ hit rate (H) (proportion of YES trials to which the participant responded YES) and false alarm rate (F) (proportion of NO trials to which the participant responded YES). The best performance would maximize H (and thus minimizes the miss rate) and minimizes F (and thus maximizes the correct rejection rate); and thus, the larger the difference between H and F, the better is the subject's sensitivity. The measure *d’* is a measure of this difference; it is the distance between the signal and the signal + noise. However, *d*’ is not simply H – F; rather, it is the difference between the *z* transforms of these two rates: *d*’ = z(H) – z(F).

When someone achieves 100% accuracy, it means they correctly identify all signal and noise trials, with no errors. In this context, the *d’* value approaches infinity because the calculation involves the difference between the z-scores of hit rates and false alarm rates. Specifically, perfect accuracy (100%) corresponds to: Hit rate (H) = 1 (or 100%), False alarm rate (F) = 0 (or 0%). Since the z-score of 1 is infinite and that of 0 is negative infinity, the *d’* in this case is theoretically infinite, reflecting perfect discriminability between signal and noise. In practice, due to the mathematical limitations of probability and z-scores, data are slightly adjusted (e.g., using a correction for perfect scores) to avoid infinite values in calculations. When H is 1, the adjustment formula used is 1 - (1 / double the number of trials), whereas when F is 0, the adjustment is 1 / double the number of trials (Hautus et al., 2021).

In our study, out of 1,024 H and F values (overall from both experiments), 60 were adjusted because participants scored 100% correct for that specific stimulus condition (the majority of these were for upright faces). Since this is a relatively simple recognition task, it is common for participants to achieve perfect scores on some trials.

We assessed performance against chance to show that both upright and inverted younger and older faces in both groups were recognized significantly above chance (For all four conditions we found *p* < .001 for this analysis). Each p-value reported for the comparisons between conditions is two-tailed, and we also report the F or t value along with effect size (η^2^p).

**Figure 1.**
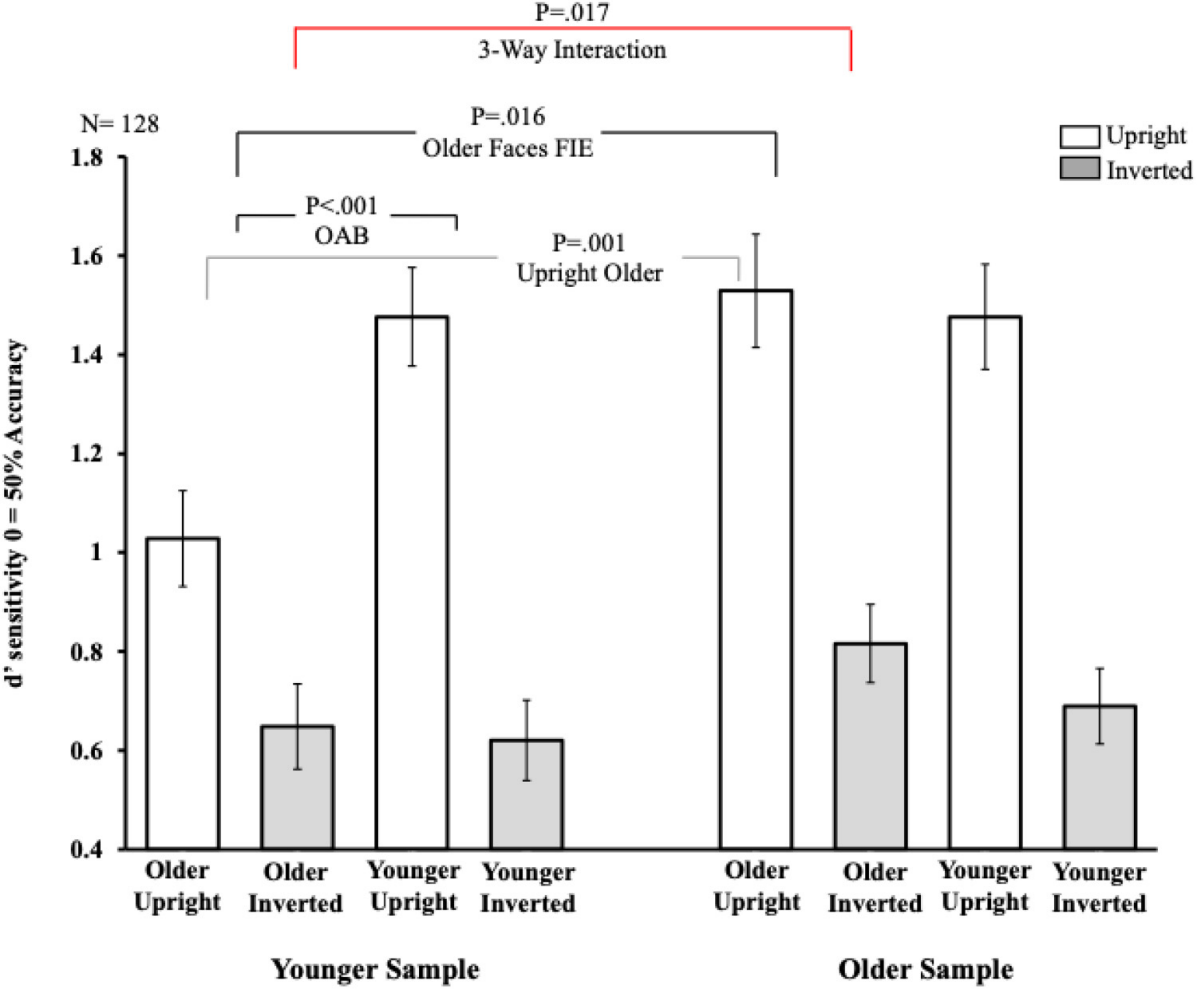
Reports the results from the study. The *x*-axis shows the face types in each sample group. The *y*-axis shows sensitivity *d'* measure. Error bars represent s.e.m.

We computed a 2 × 2 × 2 mixed model design using, as a within-subjects factor, *Orientation* (upright or inverted), and *Face Age* (younger or older) and the between-subjects factors *Participant Age* (younger or older). A mixed model Analysis of Variance (ANOVA) revealed a significant main effect of *Orientation, F*(1,126) = 161.12, *p* < .001, η^2^_p_ = .56. No significant main effects of *Face Age*, *F*(1,126) = 1.62, *p* = .20, η^2^_p_ = .01, nor *Participant Age, F*(1,126) = 3.20, *p* = .076, η^2^_p_ = .02 were found. No significant interaction was found for *Orientation *× *Participant Age*, *F*(1,126) = 1.50, *p* = .22, η^2^_p_ = .01. A significant interaction was found for *Orientation*×*Face Age*, *F*(1,126) = 10.89, *p* = .001, η^2^_p_ = .08, and the interaction *Face Age *× *Participant Age*, *F*(1,126) = 10.06, *p* = .002, η^2^_p_ = .07. Critically, the overall three-way interaction, *Orientation × Face Age × Participant Age,* was significant, *F*(1,126) = 5.83, *p* = .017, η^2^_p_ = .04 (see Figure 1). We decomposed this overall interaction by examining the two-way interactions (*Orientation *× *Face Age* i.e., the OAB) separately within each group.

**Younger Group**. A 2 × 2 ANOVA revealed a significant main effect of *Orientation, F*(1,63) = 12.79, *p* < .001, η^2^_p_ = .17, and *Face Age, F*(1,63) = 79.55, *p* < .001, η^2^_p_ = .56. Importantly, a significant interaction was also found, *F*(1,63) = 18.43, *p* < .001, η^2^_p_ = .23. Paired-sample t-tests showed a significant FIE was found for younger faces (M = .85, SD = .73), *t*(63) = 9.30, *p* < .001, η^2^_p_ = .58, and, a reduced FIE was found for older faces (M = .38, SD = .68), *t*(63) = 4.45, *p* < .001, η^2^_p_ = .24, revealing a robust OAB. An additional analysis showed that recognition for upright younger faces (M = 1.47, SD = .80) was significantly better than that for upright older faces (M = 1.02, SD = .77), *t*(63) = 5.34, *p* < .001, η^2^_p_ = .31. No difference was found between recognition performance for inverted younger faces (M = .62, SD = .65) inverted older faces (M = .65, SD = .69), *t*(63) = .36, *p* = .72, η^2^_p_ < .01.

**Older Group**. A 2 × 2 ANOVA revealed a significant main effect of *Orientation, F*(1,63) = 82.55, *p* < .001, η^2^_p_ = .57, and no significant main effect of *Face Age, F*(1,63) = 1.47, *p* = .23, η^2^_p_ = .02. No significant interaction was found, *F*(1,63) = .35, *p* = .55, η^2^_p_ < .01 indicating that the OAB was eliminated in the older group.

**Additional Analysis between groups.** We conducted an independent sample t-test which showed that the FIE for older faces was significantly larger in the older group than that found in the younger group, *t*(126) = 2.45, *p* = .016, η^2^_p_ = .09, and this was due to a higher performance for upright older faces in the older group compared to that in the younger group, *t*(126) = 3.23, *p* = .001, η^2^_p_ = .14. No significant difference was found between inverted older faces across the two groups, *t*(126) = 1.33, *p* = .19, η^2^_p_ = .03.

We then conducted the same analysis for the younger faces which revealed no significant differences between the FIE in the younger and older groups, *t*(126) = 0.51, *p* = .61, η^2^_p_ < .01. For completeness we also compared performance for upright younger faces across the two groups which revealed no significant difference, *t*(126) = 0, *p* = .99, η^2^_p_ < .01, nor did performance for inverted younger faces, *t*(126) = 0.65, *p* = .51, η^2^_p_ < .01.

## Discussion

In this study, we extended the investigation of the mechanisms underlying the OAB by examining how young and older adults recognize own-age versus other-age face stimuli. Building upon prior findings from the ORB literature (Civile & McLaren, 2022; Vizioli et al., 2010) and the OAB literature (Kuefner et al., 2008; Cassia et al., 2009), we demonstrated that the FIE can serve as a measure of the OAB, supporting the perceptual expertise account of this phenomenon. Consequently, we extended the FIE as an index of the OAB to both young and older participants.

Our results showed a robust OAB in younger participants, indicated by a significantly reduced FIE for other/older-age faces compared to the pronounced FIE for own/younger-age faces. This difference was mainly driven by significantly lower recognition performance for upright older faces versus upright younger faces supporting the lack of perceptual expertise at processing the configural/holistic information of older faces. No differences were observed between inverted older and younger faces, because on inversion, configural/holistic processing is not at play as we are not used seeing faces upside down, aligning with the perceptual expertise account (Civile, Zhao, et al., 2014; Civile et al., 2021; Diamond & Carey, 1986; Gauthier & Tarr, 1997; McLaren, 1997)

In contrast, the older group displayed a significant FIE for both own- and other-age faces, with recognition performance for upright older and younger faces being equally good Importantly, when comparing the FIE for older faces between the younger and older participant groups, a significant difference emerged due to better performance for upright older faces in the older group compared to the younger group. These findings are consistent with the perceptual expertise account: older participants, having previously been young adults, have developed experience at processing the configural/holistic information in younger faces and are currently attuned to recognizing their own-age faces. Like the younger group, older adults have no prior or current experience with upside-down faces, resulting in no differences between inverted older and younger faces.

Our results contribute to the face recognition literature on the own bias, suggesting that both ORB and OAB may share perceptual expertise mechanisms that can be measured through the FIE. For instance, Vizioli et al. (2010; for an early version of a similar paradigm see also Rhodes et al., 1989) demonstrated that both Western Caucasian and East Asian observers exhibited a reduced FIE for other-race versus own-race faces, primarily due to disrupted recognition performance for upright other-race faces. They also examined electrophysiological correlates of the OAB, showing that presentation of other-race faces resulted in a reduced FIE on the N170 ERP component latencies—a negative-polarity deflection (peak) that occurs between 130 to 210 ms after the onset of a face and is largest at parietal-occipital regions (Eimer, 2011). Future research could extend our OAB paradigm to include the investigation of electrophysiological correlates, offering further insight into the shared mechanisms underlying the own-bias phenomena.

Recently, Civile and McLaren (2022) demonstrated that a tDCS procedure, derived from perceptual learning research, could reduce the inversion effect for faces and checkerboard stimuli (Civile et al., 2021), effectively eliminating the ORB by disrupting perceptual expertise for upright own-race faces and reducing the FIE to the same level as for other-race faces. The authors proposed an explanation of the ORB within the framework of perceptual expertise manifested through perceptual learning. According to the MKM model of perceptual learning (MKM; McLaren et al., 1989; McLaren et al., 2012; McLaren & Mackintosh, 2000), exposure to prototype-defined stimulus categories facilitates perceptual learning. Initially, observers focus on common features of category exemplars, which helps associate these features with their respective categories. As this connection solidifies, the salience of common features diminishes, allowing unique exemplar features to become more prominent. This modulation of feature salience enables perceptual learning, improving the ability to discriminate and recognize upright faces. However, this advantage diminishes with inversion, due to reduced expertise with inverted faces. Factors such as specific tDCS stimulation (Civile et al., 2021), presentation of other-race faces (Civile & McLaren, 2022; Vizioli et al., 2010), induced social anxiety (Hills et al., 2019), and face manipulations like Thatcherization or scrambling (McCourt et al., 2023), all significantly reduce the FIE by disrupting recognition of upright faces. These manipulations alter salience modulation, maintaining high salience for common features and emphasizing similarities over differences, which hampers discrimination and reduces the FIE.

Future research could extend our OAB paradigm to incorporate the tDCS application used by Civile and McLaren (2022). This would test whether the OAB for younger participants might be eliminated by disrupting perceptual expertise for upright own- or younger-age faces, paralleling Civile and McLaren's findings on the ORB and providing evidence for a similar underlying mechanism.

More generally, our results also contribute to the face recognition literature by informing the ongoing debate about whether face processing primarily involves recognizing face images or extracting face identity. Currently, the research on the OAB has often employed recognition paradigms similar to those used in our study. However, no specific evidence has yet demonstrated that the OAB is a robust phenomenon when different images of the same facial identity are used, rather than the same image, to isolate the effects of image processing versus face identity recognition (Bruce & Young, 1986). Given that the FIE—which was consistently observed across both face image and face identity paradigms—serves as a reliable indicator of the OAB, our findings encourage future adaptations of this paradigm into behavioral tasks designed to address this debate. For example, tasks such as the Cambridge Face Memory Test or the Benton Face Memory Test (Duchaine & Nakayama, 2006) could be employed to potentially provide the first evidence of an OAB measured through the FIE in face identity recognition tasks.

In conclusion, our findings advance understanding of the OAB, demonstrating that younger adults show a reduced FIE for other- versus own-age faces due to disrupted recognition of upright older faces. Importantly, the results support the perceptual expertise basis of the OAB by revealing a crossover age interaction: younger participants exhibited a reduced FIE for older faces, while older participants displayed a robust FIE for the same faces. Within this single study, we provide evidence of how perceptual expertise develops across different age groups and influences face recognition.

## Supplemental Material

sj-docx-1-pec-10.1177_03010066251405714 - Supplemental material for Testing the own-age bias in face recognition among younger and older adults via the Face Inversion Effect Supplemental material, sj-docx-1-pec-10.1177_03010066251405714 for Testing the own-age bias in face recognition among younger and older adults via the Face Inversion Effect by Ciro Civile and Guangtong Wang in Perception
