# Supplementary material for: Testing the own-age bias in face recognition among younger and older adults via the face inversion effect
Source: Perception. 2025 Dec 15;55(6):544–56. doi: 10.1177/03010066251405714 (PMC13191059; doi:10.1177/03010066251405714)
Supplement: sj-docx-1-pec-10.1177_03010066251405714 - Supplemental material for Testing the own-age bias in face recognition among younger and older adults via the Face Inversion Effect [file sj-docx-1-pec-10.1177_03010066251405714.docx]

**Testing the Own-Age Bias in face recognition among younger and older adults via the Face Inversion Effect**

Ciro Civile and Guangtong Wang

Department of Psychology, Faculty of Health & Life Sciences,

University of Exeter, UK

**Results: Percentage Correct (%) analysis**

We conducted the same statistical analysis as in the main manuscript, this time using percentage correct scores. (%) The results confirmed the effects previously identified with *d’.*

The ANOVA (*Orientation* x *Face Age* x *Participant Age*) revealed a significant main effect of *Orientation, F*(1,126) = 161.39, *p* < .001, η^2^_p_ = .56. No significant main effects of *Face Age*, *F*(1,126) = .81, *p* = .37, η^2^_p_ < .01, nor *Participant Age, F*(1,126) = 3.46, *p* = .067, η^2^_p_ = .02 were found. No significant interaction was found for *Orientation* x *Participant Age*, *F*(1,126) = 2.59, *p* = .11, η^2^_p_ = .02. A significant interaction was found for *Orientation* x *Face Age*, *F*(1,126) = 10.46, *p* = .002, η^2^_p_ = .08, and the interaction *Face Age* x *Participant Age*, *F*(1,126) = 9.36, *p* = .003, η^2^_p_ = .07. Critically, for % as well, the three-way interaction was significant, *F*(1,126) = 4.20, *p* = .042, η^2^_p_ = .03 (see Figure 2). We decomposed this overall interaction by examining the two-way interactions (*Orientation* x *Face Age* i.e., the OAB) separately within each group.

**Younger Group**. A 2 x 2 ANOVA revealed a significant main effect of *Orientation,* *F*(1,63) = 71.78, *p* < .001, η^2^_p_ = .53, and *Face Age, F*(1,63) = 9.13, *p* = .004, η^2^_p_ = .13. A significant interaction was also found, *F*(1,63) = 13.81, *p* < .001, η^2^_p_ = .18, led by a larger a larger FIE for younger faces (M=12, SD = 11.7), *t*(63) = 8.39, *p* < .001, η^2^_p_ = .53, compared with the FIE found for older faces (M=6, SD =11.1), *t*(63) = 4.10, *p* < .001, η^2^_p_ = .21, confirming a robust OAB. Recognition for upright younger faces (M=73%, SD=12.7) was significantly better than that for upright older faces (M=66%, SD=11), *t*(63) = 4.70, *p* < .001, η^2^_p_ = .26. No difference was found between recognition performance for inverted younger faces (M=.60%, SD=11) inverted older faces (M=.61%, SD=11.4), *t*(63) = .46, *p* = .64, η^2^_p_ < .01.

**Older Group**. A 2 x 2 ANOVA revealed a significant main effect of *Orientation,* *F*(1,63) = 89.64, *p* < .001, η^2^_p_ = .59, and no significant main effect of *Face Age, F*(1,63) = 2.04, *p* = .16, η^2^_p_ = .03. No significant interaction was found, *F*(1,63) = .71, *p* = .40, η^2^_p_ = .01 indicating that the OAB was eliminated in the older group.

**Additional Analysis between groups.** We conducted the same analysis as those on the manuscript, which confirmed that the FIE for older faces was significantly larger in the older group than that found in the younger group, *t*(126) = 2.32, *p* = .021, η^2^_p_ = .08, and this was due to a higher performance for upright older faces in the older group compared to that in the younger group, *t*(126) = 3.33, *p* = .001, η^2^_p_ = .15. No significant difference was found between inverted older faces across the two groups, *t*(126) = 1.20, *p* = .23, η^2^_p_ = .02.

We then conducted the same analysis for the younger faces which revealed no significant differences between the FIE in the younger and older groups, *t*(126) = 0.02, *p* = .97, η^2^_p_ < .01.

**Figure 2** reports the results from the study (%). The *x*-axis shows the face types in each sample group. The *y*-axis shows %. Error bars represent s.e.m. We assessed performance against chance to show that both upright and inverted younger and older faces in both groups were recognized significantly above chance (For all four conditions we found *p* < .001 for this analysis).
